# Photon-Induced Photo-Acoustic Streaming vs. Shock Wave-Enhanced Emission Photo-Acoustic Streaming—The Effect of Three Final Irrigation Protocols on the Bond Strength of an Individually Formed Fiber Post

**DOI:** 10.3390/dj12080237

**Published:** 2024-07-26

**Authors:** Cassandra Lupita, Daliana Emanuela Bojoga, Alessandro Del Vecchio, Dan Ioan Stoia, Ion Grozav, Mariana Ioana Miron, Darinca Carmen Todea

**Affiliations:** 1 Department of Oral Rehabilitation and Dental Emergencies, Faculty of Dentistry, Victor Babes University of Medicine and Pharmacy, 300041 Timisoara, Romania; cassandra.lupita@umft.ro (C.L.); miron.mariana@umft.ro (M.I.M.); todea.darinca@umft.ro (D.C.T.); 2 Interdisciplinary Research Center for Dental Medical Research, Lasers and Innovative Technologies, 300041 Timisoara, Romania; 3 Department of Oral and Maxillofacial Sciences, Sapienza University of Rome, 00161 Rome, Italy; alessandro.delvecchio@uniroma1.it; 4 Department of Mechanics and Strength of Materials, Faculty of Mechanical Engineering, Polytechnic University of Timisoara, 300222 Timisoara, Romania; dan.stoia@upt.ro (D.I.S.); ion.grozav@upt.ro (I.G.)

**Keywords:** LASER, SWEEPS, PIPS, fiber post, endodontics

## Abstract

(1) Background: This study aimed to evaluate how laser-activated irrigation (LAI) influences the retention of a fiber post when used before an endodontic filling, as well as after post space preparation. (2) Materials and Methods: Sixty freshly extracted human incisors were selected. The teeth were randomly assigned to three groups—CONVENTIONAL (CONV), PIPS or SWEEPS—and treated endodontically. Each group received irrigation with 1 × 5 mL EDTA (17%) and 3 × 5 mL NaOCl (5.25%). In the first group, the irrigants were not activated, while in the second and third group, LAI was adopted using PIPS and SWEEPS protocols (Lightwalker from Fotona, Ljubliana, Slovenia). After post space preparation, each group received the same irrigation protocol initially established. Sticky posts (everStick Post, GC AUSTRIA GmbH Swiss) were individually adapted to the corresponding post spaces and cemented using dual cure resin cement (Gradia Core, GC Austria GmbH Swiss). All specimens were vertically embedded into self-curing acrylate (Duracryl plus, Spofa Dent, Europe), and each was sectioned into three segments of type A and type B samples for debonding through push-out and pull-out tests. The results were statistically analyzed. (3) Results: The pull-out test showed the superiority of the SWEEPS group, with a mean fracture force of 133.0 ± 50.7 N, followed by the PIPS group, with 102 N, with a lower standard deviation of ± 34.5 N. The CONV group registered the lowest fracture force. Concerning the push-out test, the SWEEPS group showed superior shear stress in comparison to the other two groups (13.45 ± 4.29 MPa); the CONV group was inferior, with shear tension values of 8.31 ± 4.67 MPa. (4) Conclusions: It can be stated that the SWEEPS and PIPS protocols resulted in considerably higher fiber post retention than the conventional method, whereas the SWEEPS protocol was superior to the PIPS protocol.

## 1. Introduction

Currently, minimally invasive dentistry is the main goal of daily dental practice. When restoring an endodontically treated tooth presenting considerable structural loss, the use of fiber posts is inevitable and critical to ensuring the longevity of a tooth restoration. The problem with endodontically treated teeth is that very often little remains of the dental structure [1,2]; at the same time, the physical properties of devitalized teeth are very different from those of vital ones [3,4], as they are brittle and less resistant [4]. To increase fracture resistance in these cases, the use of fiber posts is essential [5,6,7].

Fiber posts permit a conservative restoration of the tooth and an acceptable aesthetic result [8]. However, these restorations have issues arising from the fixation of the post, relating to many factors. Among these factors, the elements involved in post retention are the dentin surrounding the post, the luting cement used, the type of post, and the surface conditions. Those factors can play an important role in adhesion of a fiber post, which may result in success or failure of the treatment [8]. These failures often result in the fracture of the tooth, which may lead to its loss [8]. However, it is well known that the main cause of fiber post failure is related to debonding [8,9,10,11,12,13].

The condition of the root canal dentin significantly contributes to the adhesion of a fiber post. In fact, many restorations using fiber posts fail due to the difficulties in achieving proper adhesion to dentin [14]. One of the biggest limitations of these procedures is the lack of proper removal of the primary and secondary smear layers. Moreover, the surface morphology of the intracanal dentin is also of great importance. These two factors are closely related to the type of irrigant and its activation during the root canal cleaning and shaping phases.

Conventional endodontic procedures involve different mechanical instruments and disinfection agents, leading to several limitations and disadvantages that are associated with the complexity of the root canal system, such as difficult-to-remove debris and disinfection. The smear layer is often tenacious, and in 30% of the canal surfaces, it is not removed properly [15]. This means that the dentinal tubules are covered, thus making the penetration of the irrigant impossible, leading to limited antibacterial action in terms of conventional irrigation [16]. Many studies have shown that the activation or agitation of the irrigant plays a key role in minimizing these deficiencies [17,18,19,20]. Over the years, many activation systems for endodontic irrigation have been introduced and studied.

Recently, the application of lasers has been introduced, resulting in the so-called laser-activated irrigation (LAI). In many studies, this method has demonstrated increased bond strength between the fiber post and dentin [21,22,23]. LAI involves the use of radial-firing tips to improve the lateral emission of photons that irradiate the irrigants [24]. Among these tools, many studies have revealed the high efficiency of photon-induced photo-acoustic streaming (PIPS) and, more recently, shock wave-enhanced emission photo-acoustic streaming (SWEEPS), which, through the use of the first and second cavitation effect, improve the chemical activation of the irrigants and the subsequent removal of the smear layer [25,26,27,28]. The use of these methods leads to greater physical decomposition and disruption of biofilms [29] and a greater bactericidal effect and provides effective fluid flow, especially in narrow canals [26]. LAI has a triple-level effect: a bactericidal effect, a morphological effect on the intracanal dentin walls, and irrigant activation [24]. Furthermore, PIPS reduces thermal effects and exerts both stronger cleaning and bactericidal action [30,31]. Therefore, the Er:YAG laser is a very promising tool for overcoming the numerous limitations of conventional irrigation protocols [32]. Through the generation of a high power peak, the primary phenomena of an explosion and secondary cavitation are created. This happens at a significant distance from the tip of the fiber placed in the pulp chamber, where it is activated. In this way, it generates an irrigant flow speed that is ten times higher than that of conventional ultrasonic passive irrigation [24,33]. In the SWEEPS protocol, two laser pulses are used [24]. The advantage is that we can time the second laser pulse in a way that it coincides with the moment the first bubble starts to collapse. Due to the second laser pulse, a second, new bubble is formed, and the pressure rises even more, thus accelerating the implosion of the first bubble. This pressure boost creates a high shock wave which, at the same time, determines higher sheer stresses in the closed chamber space that is the root canal system. Even though the SWEEPS protocol was recently developed, much research has been undertaken to analyze and understand its advantages. In general, the activation of the irrigant using the Er:YAG laser via the PIPS and SWEEPS methods shows several advantages in comparison to conventional irrigations systems and other types of LAI, as follows: highest chemical activation of irrigants, especially sodium hypochlorite (NaOCl); greater removal of the smear layer compared to that obtained through the use of EDTA; important chemical disintegration of remaining pulp residues via NaOCl; greater physical decomposition or disruption of the biofilm inside the root canal, and subsequently, a greater bactericidal effect; and a more effective flow of the fluids, which is also the case in narrow canals, with a much faster speed in the direction of the apex that uses less pressure due to the Venturi effect, which is beneficial to calcified canals and in the removal of broken instruments from the root canal [24]. EDTA and sodium hypochlorite are common irrigants that work very well [34], not only in combination with LAI. NaOCl has high bactericidal effects and the ability to remove residual organic tissue, but used alone, it is not able to properly remove the smear layer, hence the recommendation of its application in association with EDTA [35]. Studies have shown that activated irrigation using an Er:YAG laser and 17% EDTA for a duration of 20 s revealed an uncovered collagen matrix, the absence of a smear layer, the opening of dentinal tubules, and an absence of debris [24]. In this context, the superior removal of the smear layer also enables greater success in restoring teeth with the application of fiber posts. Moreover, due to the fact that the tip is lightly positioned into the pulp chamber, it also enables minimally invasive root canal shaping [28] not only for the cleaning of the endodontic space but for the preparation of the fiber post space [33]. The size of the root canal does not have to be much enlarged to obtain good debridement [33]; thus, less dental tissue loss means greater stability for the tooth structure, decreasing the risk of tooth fracture after fiber post placement and endodontic retreatments.

*Purpose of the study.* This study aimed to evaluate how laser-activated irrigation (LAI) influences the retention of a fiber post when used before an endodontic filling, as well as after post space preparation. At the beginning of the study, the null hypothesis was that there would be no statistically significant differences among the three experimental study groups.

## 2. Materials and Methods

### 2.1. Study Design

The study was performed at the School of Dentistry, University of Medicine and Pharmacy “Victor Babes”, Timisoara, in collaboration with the Polytechnical University of Timisoara, Department of Material Engineering and Manufacturing, Romania. The experimental study design was approved by the Local Ethics Committee (no 48/12 September 2019).

Sixty freshly extracted single root teeth were selected and endodontically prepared within 48 h from the moment of extraction. The teeth were extracted for periodontal reasons from different patients. The inclusion criteria were the absence of carious lesions and previous endodontic treatments as well as the absence of defects resulting from surgery, one single root canal and root canals without very pronounced angulations. After this, an X-ray examination was performed, and all the teeth that did not correspond to the inclusion criteria were removed. Therefore, only 48 teeth were included in the subsequent experiments.

During the whole experiment, the teeth were kept in saline solution that was changed periodically to avoid desiccation.

### 2.2. Preparation of the Specimens

The specimens were cleaned via ultrasonic scaling and professional dental brushing using a non-fluoridated paste so that the specimens were free from residual tartar deposits and periodontal residuals. After that, each specimen was administered an X-ray examination. The crowns of those teeth were sectioned 2 mm coronal from the cement–enamel junction (CEJ) using burs.

### 2.3. Endodontic Treatment of the Specimens

Access to the root canal was created and lightly widened. Then, cleaning and shaping were performed using hand files up to ISO 15, continuing with the preparation of the root canal using Reciproc Blue files (VDW Dental GmbH, München, Germany), using instrument size R 25. Then, final irrigation was carried out. For the CONV group, EDTA and NaOCl irrigant was used without laser activation. In the PIPS and SWEEPS groups, the LAI was performed with the help of Er:YAG laser (Light Walker Er:YAG laser, Fotona, Ljubliana, Slovenia) with the PIPS tip or the SWEEPS tip and the H14 handpiece. Irrigating three times with 5 mL distilled water was the final step.

The specimens were randomized into three groups:

- CONVENTIONAL group: conventional root canal and dentin of the post space irrigation using 1 × 5 mL EDTA 17% and 3 × 5 mL of 5.25% NaOCl without activating the irrigant. After each irrigant, 5 mL of distilled water was used.
- PIPS group: laser-assisted irrigation of the root canal and dentin of the post space using PIPS protocol with 3 × 5 mL of 5.25% NaOCl, 5 mL distilled water, 1 × 5 mL EDTA 17%, and finally, 5 mL of distilled water.
- SWEEPS group: laser-assisted irrigation of the root canal and dentin of the post space using the SWEEPS protocol with 3 × 5 mL of 5.25% NaOCl, 1 × 5 mL EDTA 17%, and finally, 5 mL of distilled water.

The following irrigation activation protocols were used:

- Conventional irrigation using syringe irrigation, without activation;
- LAI PIPS: 20 mJ, 15 Hz, 40 s (H14 handpiece), 1 cycle EDTA, and 3 cycles NaOCl;
- LAI SWEEPS: 20 mJ/repetition rate, 15 Hz, 30 s (Varion 400 μm), 1 cycle EDTA, 3 cycles NaOCl.

The PIPS tip and SWEEPS tip were lightly placed in the pulp chamber of the extracted tooth for both the final irrigation of the endodontic treatment and the final irrigation of the prepared post space. Under constant syringe irrigation, laser firing was undertaken. Right after the first laser shot in the PIPS group, it was noticed that the irrigant flushed out by the laser was opaque and muddy for a second, but then it turned clear. After finishing the last round of irrigation, the root canal was dried with paper points, and endodontic filling commenced with lateral condensation and vertical compaction. AH Plus (Dentsply, Sirona, Charlotte, NC, USA) was used as the sealant. Afterward, the gutta-percha point was cut with an ultrasonic system, thus finishing the root canal treatment. The root canal filling was left to harden for 48 h.

### 2.4. Post Space Preparation

After 48 h, the post space preparation for the fiber posts was carried out using a 1.3 mm digging bur for the small teeth and a 1.6 mm digging bur for the bigger teeth, using water and air for cooling. After measuring the root canal beforehand, 2/3 of the root canal filling was removed. After that, the irrigation of the post space was completed, as shown above, using 5 mL of EDTA for the removal of the second smear layer and 5 mL NaOCl five times, completed with the conventional etching using 35% H_3_PO_4_. For the second group, the PIPS protocol was used, and for the third group, the SWEEPS protocol was used, with the same irrigation protocol used for endodontic treatment.

### 2.5. Cementation of the Individually Formed Fiber Post

Immediately after the procedure, the post space was dried using paper points and each sticky post was adapted to the post space using different diameters: 1.2, 1.3, and 1.6. The different diameters were combined for better adaptation to the different tooth sizes and post spaces, and the cementation procedure commenced according to the instructions. In this study, Ever Stick Post and stick resin (GC Austria, Gratwein-Strassengel, Austria) were used. Gradia Core (GC Austria, Gratwein-Strassengel, Austria) dual-cured radiopaque for core buildup and post cementation was used as the resin for cementation. At the end of each cementation, the specimens were immersed into a saline solution.

### 2.6. Embedding of the Specimens

The specimens were vertically embedded into boxes with identical dimensions. The samples were placed on a vibrating table to ensure the regular distribution of the self-curing acrylate (Duracryl plus, SpofaDental), which was prepared according to the producer’s instructions, and inserted into the boxes, after which the specimens were inserted into the acrylate.

### 2.7. Push-Out and Pull-Out Tests

**Figure 1:**
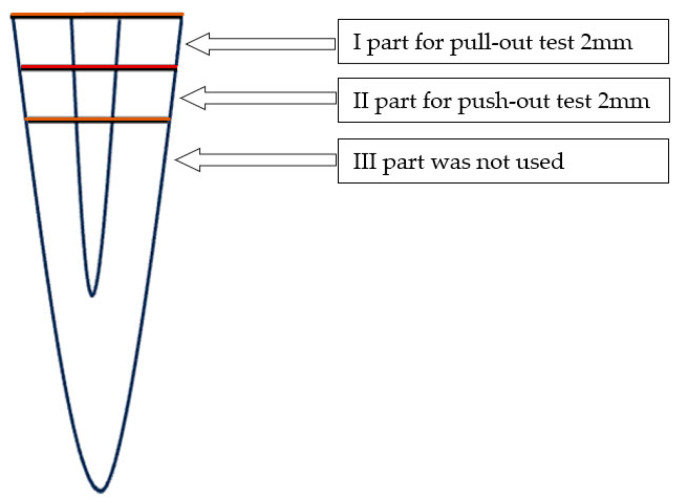
The sectioned parts of the root considered in this study.

The testing conditions were as follows: each root of all groups (CONV, PIPS group, and SWEEPS group) was horizontally sectioned to obtain samples of the same size (4 mm) for both the pull-out test A type and the push-out test B type. Each 4 mm was divided into slices of 2 mm. The residual parts of the sectioned specimens (corresponding to the third apical part) were not considered. Finally, 96 specimens (48 samples for pull-out test—slices of the coronal part; 48 samples for push-out test—slices from the middle third of the roots) were considered, which were divided into two principal groups, A and B, which were allocated to two different tests, respectively, pull-out tests (t A) and push-out tests (t B) (Table 1, Figure 1).

The number of groups consisted of three A-type and three B-type samples, and the number of samples per group was 16. The type of loading was represented by the pull-out and push-out of the fiber post through the dental structure; Mecmesin Multitest 5i was used, and device control was achieved via displacement.

**Figure 2:**
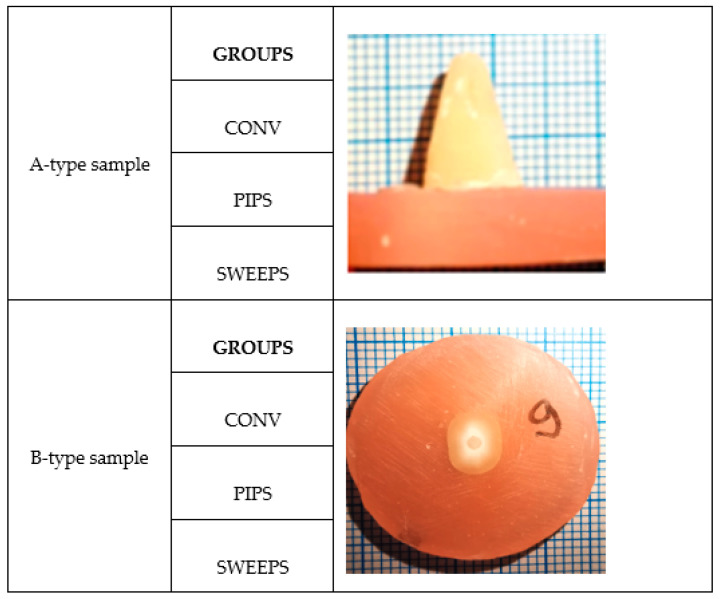
The aspects of sample types A and B for all 3 groups.

Sample types:

A: the sample is loaded by pulling the fiber post from the dental structure (positive axial loading causing shear stress at the interface) (Figure 2).

B: the sample is loaded by pushing out the fiber post through the dental structure (negative axial loading causing shear stress at the interface).

**Figure 3:**
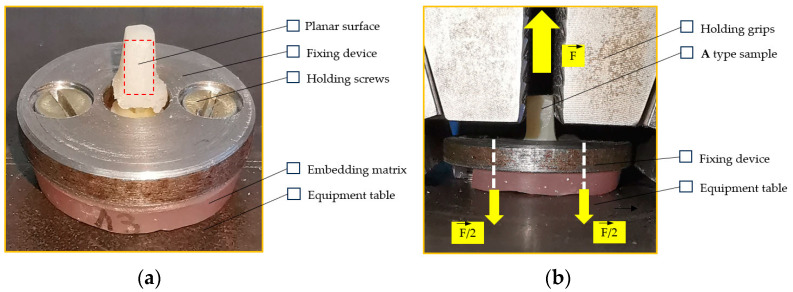
The scheme of sample loading: (**a**) sample mounting into the fixing device; (**b**) sample gripping, ready for test.

Type A samples:

The fixing device was manufactured to allow for the embedding material to remain rigidly secured to the equipment table, as well as for the fiber post to remain available for gripping (Figure 3). To ensure repeatability in terms of gripping, the reconstructed posts were faceted by grinding, and the embedding matrix was drilled to permit screw fixation on the machine table.

**Figure 4:**
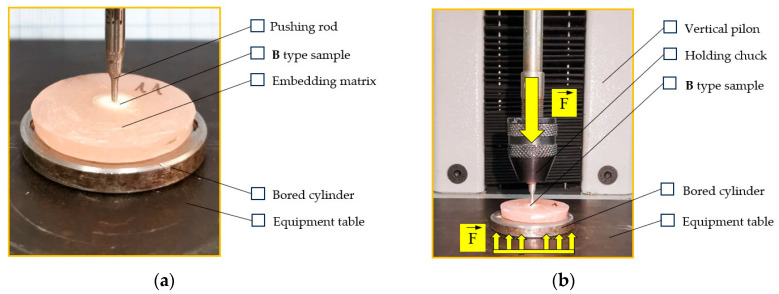
The type B sample loading: (**a**) sample sustaining piece; (**b**) testing equipment.

Type B samples:

The type B samples are characterized by a well-determined fiber post height in the dental structure and by the cross-section of the fiber post. The geometrical characteristics of the sample and the force–displacement curves enable the determination of the shear stress that occurs at the adhesive interface between the fiber post and the reconstruction material. In Figure 4, the testing pin and the structure fixing are presented. The sample is sustained by a bored piece that allows the expulsion of the fiber post and prevents the pure compression of the post.

The results were statistically analyzed by applying analysis of variance with a Tukey–Kramer test, the Fisher method, and Hsu’s MCB method for multiple comparisons using Minitab 16 software (https://www.minitab.com/en-us/products/minitab/, accessed on 1 February 2024), by Microsoft. The results are presented in dedicated sections.

## 3. Results

**Figure 5:**
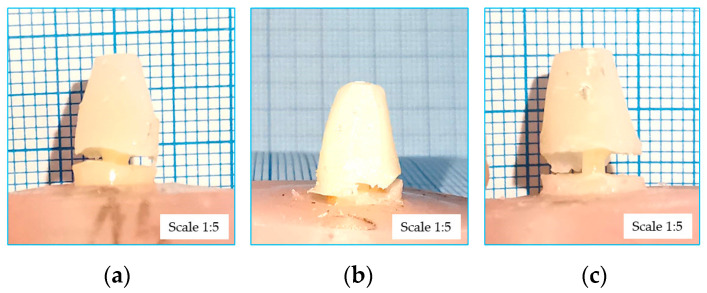
The sample breaking by pulling: (**a**) PIPS group; (**b**) SWEEPS group; (**c**) CONV group.

Type A samples:

During the mechanical tests, the force–displacement curves for each A sample of the three groups were obtained. In Figure 5, the representative failure images of each group can be observed, all presenting the pull out of the post from the restoration materials and no failure of the post itself.

The representative curves were recorded until the final failure of the structure using a sampling frequency of 100 Hz. The linear segment corresponding to a displacement of up to 0.4 mm signifies the elastic behavior of the structure, which was followed by elastic–plastic transition up to the final failure. As an initial assessment, significantly higher pulling forces are observed in the PIPS and SWEEPS groups compared to those of the CONV group; nevertheless, when comparing the force values with the actual size and properties of the individual samples, the results are quite complex. Therefore, the sample B type will give a much better approach.

For an improved visualization and interpretation of the pulling results, the maximum forces for each sample of each group were identified and arranged, showing the superior mechanical behavior of the PIPS and SWEEPS groups in comparison with the CONV. group.

The mean and standard deviation of each group were computed. Here, the superiority of the SWEEPS group shows a mean fracture force of 133.0 ± 50.7 N, followed by the PIPS group, with a mean fracture force of 102 N but with a lower standard deviation of ± 34.5 N. Considering the means and standard deviations, we can conclude that SWEEPS and PIPS groups have comparable mechanical behavior. On the other hand, the CONV group has the lowest fracture force (Table 2).

**Figure 6:**
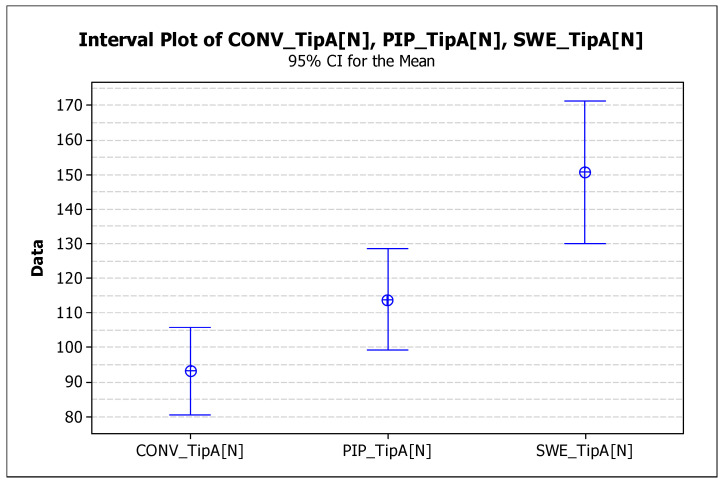
Statistical results of the pull-out test for Type A samples in the CONV, PIPS, and SWEEPS groups.

The statistical results of the pull-out test can be observed below (Figure 6).

The SWEEPS_A interval does not overlap with the other intervals; therefore, it is significantly different from CONV_A and PIPS_A. The PIPS_A interval overlaps CONV_A; it does not differ significantly from CONV_A.

PIPS_A is not significantly different from CONV_A (zero is included in the range). SWEEPS_A is significantly different from CONV_A (zero is not included in the range)

There are significant differences between SWEEPS_A, CONV_A, and PIPS_A. Zero is included in the range. Significant differences were recorded between CONV_A and PIPS_A.

Zero was included in the PIPS_A_CONV range, and there are no significant differences between PIPS_A and CONV_A. This is not included in the SWEEPS_A_CONV interval, and there are significant differences between SWEEPS_A and CONV_A.

Zero in the PIPS_A_CONV interval; no significant differences were found between PIPS_A and SWEEPS_A. Zero is not in the SWEEPS_A_CONV range, and significant differences were observed between SWEEPS_A and CONV_A.

**Figure 7:**
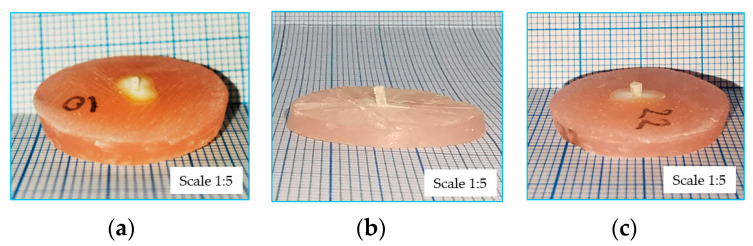
Sample failures due to pushing of the fiber post: (**a**) PIPS group; (**b**) SWEEPS group; (**c**) CONV. group.

Type B samples:

After mechanical testing, the force–displacement curves for every type B sample of the three groups were obtained. Figure 7 presents representative images of sample failures for each group. In each case, it can be observed how the expulsion of the fiber post from the dental structure through the fracture of the interface took place.

Using the relationship outlined below, the shear stress (*τ*) was computed using the maximum force values at the fracture point (*F*_max_) and the lateral area of the cylindrical shape of the fiber post, with the geometric characteristics *R* and *h*.

$$\tau =\frac{F}{A}=\frac{F_{\max}}{2\cdot \pi \cdot R\cdot h}\left[N/{\mathrm{mm}}^{2}\right]$$

**Figure 8:**
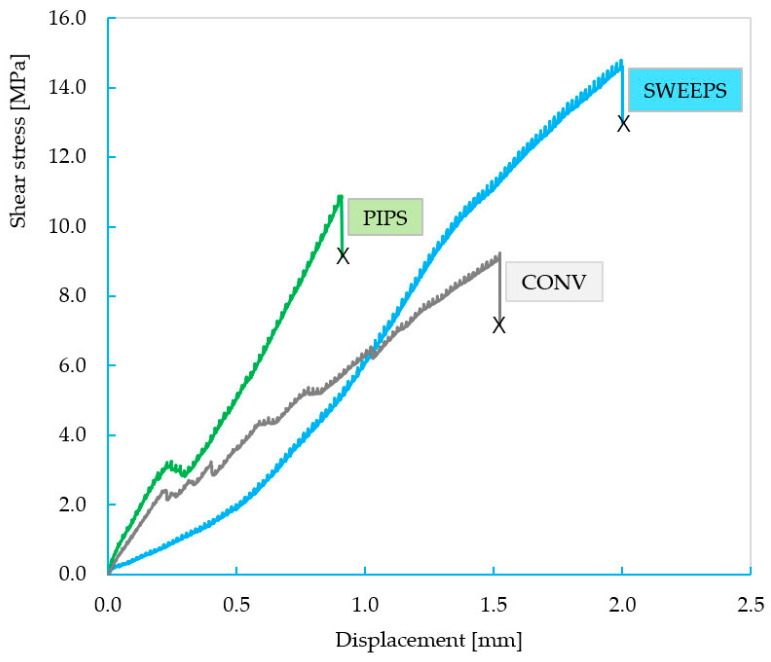
The characteristic force–displacement curves of the CONV, PIPS, and SWEEPS groups.

The stress–displacement characteristic curves for these testing samples are presented in Figure 8. The shear stress was computed as mentioned, while the displacement was achieved via computation.

**Figure 9:**
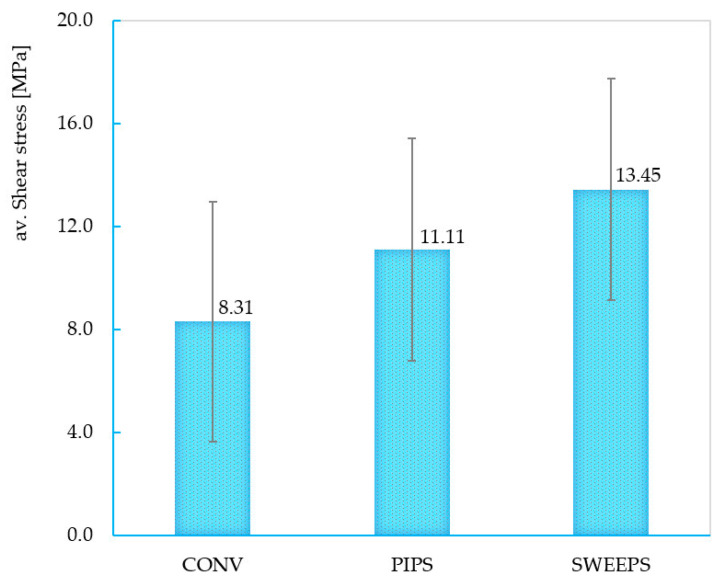
Chart of the mean and standard deviation of shear stress.

The characteristic curves were recorded until the final failure of the structure, as in the previous case. This time, much better linearity in the curves is observed due to the better geometrical control of the structure. Also, from the stress–displacement curves, it can be observed that the SWEEPS group is superior to the other two groups; conversely, the CONV group is inferior. Figure 9 shows the mean and standard deviation of the stress values of each group. It can be observed that SWEEPS reaches superior shear stress in comparison to the other two groups (13.45 ± 4.29 MPa); conversely, the CONV group is inferior, with shear tension values of 8.31 ± 4.67 MPa (Table 3).

**Figure 10:**
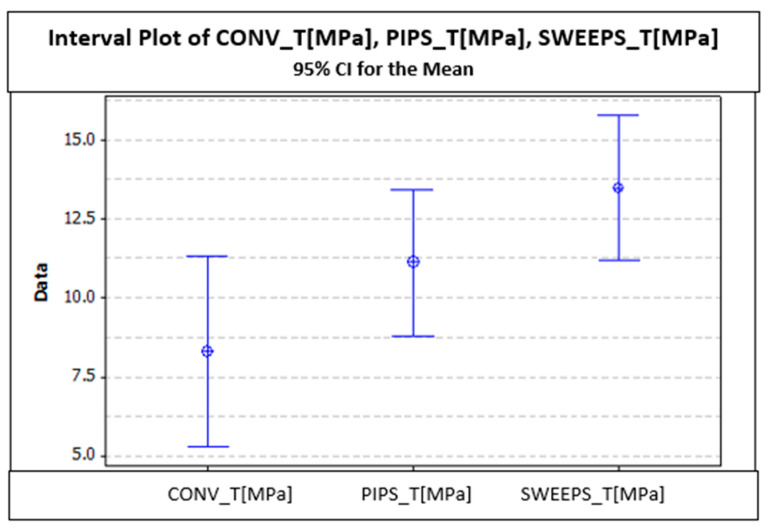
Graphical results of the pull-out test for Type B samples in the CONV, PIPS, and SWEEPS groups.

Figure 10 shows a graphical interpretation of results for the shear test.

There are significant differences in terms of *p* < 0.05 (5%). According to different types of statistical analysis (ANOVA, Hsu’s multiple comparison test, Tukey and Fisher tests), there are significant differences between SWEEPS_T and CONV_T. There are no significant differences between SWEEEPS_T and PIPS_T; PIPS_T and CONV_T, respectively.

Because the value of strain up to failure differs among the groups but also within the group, the breaking energies were calculated. This parameter was computed as the surface area beneath the stress–displacement curve and indicates the mechanical energy required to fracture the structure. The breaking energy is superior in the case of SWEEPS_T, showing not only higher absolute force but also superior toughness.

It can be considered that there are almost significant differences between SWEEPS_T and CONV_T; zero is included very little in SWEEPS_T (-0.095). Significant differences were recorded between PIPS_T and CONV_T.

There are no significant differences between PIPS_T and CONV_T (zero is included in the range). There is a significant difference between SWEEPS_T and CONV_T (zero is not in range).

## 4. Discussion

The present study confirms that laser-assisted irrigation increases the retention of an individually formed fiber post to the dentin of the post space, therefore, yielding better results than the conventional method of syringe irrigation. Based on these results, the null hypothesis was rejected.

This study aimed to evaluate the manner in which the PIPS and SWEEPS protocols developed by Fotona influence the retention of a fiber post compared to conventional irrigation methods. Implicitly, the present study allowed us to compare the PIPS and SWEEPS protocols.

Statistical analyses using one-way ANOVA demonstrated that there are significant differences among the three groups (CONV, PIPS, and SWEEPS), with *p* < 0.05 in both protocols: pull-out (Type-A) and push-out (Type-B).

The data analysis confirmed the superiority of the SWEEPS protocol in contrast to the PIPS protocol; in fact, this finding was also confirmed by a study published in 2017 by N. Lukac et al., which also states that “the use of SWEEPS promises to significantly enhance the efficacy of the standard PIPS laser-induced irrigation procedures” [36]. To date, conventional endodontics has made big steps forward. The only limitation of this method is linked to imperfect decontamination of the root canal. One of the biggest advantages and the main reason the use of this laser technique is superior is that it provides an almost ideal decontamination of the root canal [37]. In this day and age, the PIPS and SWEEPS methods show clear value when it comes to endodontic treatments; in fact, the introduction of the PIPS and SWEEPS protocols has significantly increased the quality of root canal decontamination. The great advantage of using a laser is based on bacterial reduction, which is very important in enhancing bond strength when compared to conventional methods [38,39]. Moreover, the correct preparation of the root canal in view of fiber post placement continues to be a key element in the stability of corono–radicular reconstruction, which is an integral part of extended multiunit fixed prosthesis [39]. In this study, we mainly focused on the connection between intracanalar dentin and a fiber post. For future studies, our efforts will focus on covering the relationship between corono–radicular restoration and future multiunit fixed prosthesis in comparison with single-unit crowns.

The push-out test has been used in numerous studies, most frequently in conjunction with the microtensile testing method. It is the recommended procedure [37]. Studies show that the push-out test is the most reliable and precise method for testing the retention of an individually formed fiber post [31,38]. That is why we used this testing method in our in vitro study and, for the first time, we evaluated the effect of modifying the intracanalar surface using the PIPS and SWEEPS methods versus conventional techniques. In this study, we divided the teeth into A and B samples and additionally performed a pull-out test. As irrigants for this study, we used sodium hypochlorite and EDTA, which are the two preferred irrigants currently used. Furthermore, many studies have shown that this combination of irrigants could be very advantageous regarding the retention of a fiber post [23,28,34,39,40,41,42,43]. However, studies have also shown that the use of CHX as an irrigant can be promising as well, meaning that it is suitable for inclusion in future studies [44].

During the study, all of the teeth were kept in saline solution to avoid desiccation. However, this could constitute a possible limitation, as it is very difficult to imitate the same conditions we find in the oral cavity. These conditions include the hygiene of the oral cavity, the presence of saliva, occlusal stability and its momentary condition, and physiological odder pathological abrasion [45,46,47,48].

The use of individually formable Ever Stick Posts from GC seems to have been a good choice as their initial flexibility permits good adaptation to the different sizes of post spaces, permitting a more conservative preparation of the post space. Also, studies show that individually formed fiber posts demonstrate a higher bond strength than the prefabricated ones [49,50].

For the A samples, we first collected the representative failure images of each group after each pull-out test. These images depicted the sample breaking due to pulling, but no failure of the post itself was observed. The initial assessment showed significantly higher pulling forces in the PIPS and SWEEPS groups compared to the CONV group. To provide a better general evaluation, we used upper as well as lower incisors. Although we did our best to choose similar tooth sizes and shapes, correlating the force values with the actual size and properties of the individual samples was difficult. At this point, the samples of the B type will lead to a better approach because of the well-determined height of the fiber post involving the dental structure and due to the cross-section of the fiber post. The superiority of the SWEEPS group as opposed to PIPS is illustrated by the different fracture forces. SWEEPS has the highest value of 133.0 ± 50.7 N and the CONV group has the lowest value of ±34.5 N. The PIPS group had a value of 102 N. When considering the means and standard deviations, it leads to the conclusion that the SWEEPS and PIPS groups show comparable mechanical behavior. Statistical analyses using one-way ANOVA demonstrate that there are significant differences among the three groups (CONV, PIPS, SWEEPS) with *p* < 0.05.

The type B samples allowed for the determination of the shear stress that happens at the adhesive interface between the fiber post and the reconstruction material. Here, sample failure is represented by the expulsion of the fiber post due to the fracturing of the interface. Therefore, due to the better geometrical control of the structure, a much better linearity of the curves can be observed. Also, in this case, the superiority of the SWEEPS group in terms of shear stress can be observed, with a value of 13.45 ± 4.29 MPa; the CONV group is inferior to the other two, with a value of 8.31± 4.67 MPa.

The breaking energy is also superior in the case of SWEEPS as it shows a higher absolute force and superior toughness.

A very recent study from T.C. Bohrer et al. clearly showed that treating the post space with the help of laser and NaOCl has a positive effect on the bond strength of a fiber post [39]. He also included the Nd:YAG and Er,Cr:YSGG lasers. In fact, numerous studies have evaluated the effect of using an Er,Cr:YSGG laser on the retention of fiber posts [47]. The results of the studies are split, with some of them showing the positive effect of Er,Cr:YSGG on increasing the bond strength [13,48,51]; however, others suggest the opposite [52,53,54], and when compared to the use of PDT, F.A. Alonaizan et al. suggested that Er,Cr:YSGG was inferior to PDT [55,56].

A study conducted by F. Alshammary et al. compared the effect of PDT (photodynamic therapy), Nd:YAG and Er,Cr:YSGG on the push-out test, with their results showing that from all three groups, PDT was superior to the other two [48,55]. Other studies have compared LAI, ultrasonic agitation, and conventional syringe irrigation, also demonstrating the superiority of LAI [13,22,57,58].

According to the results contained within the specialized literature mentioned above, in which the Er,Cr:YSGG and Nd:YAG lasers proved their effectiveness in increasing the adhesion of fiber posts to the root structure of the tooth; the same increase in adhesive phenomenon was also recorded after the application of the Er:YAG laser in SWEEPS mode. Additionally, in this study, the Er:YAG laser was initially applied as a method of decontaminating the root canal in order to obtain more permeable dentin with minimal microleakage, an aspect that influences the degree of adhesion of the adhesives to the dentinal structure. By minimizing microleakage with the Er:YAG laser, the adhesion achieved was clearly superior in the SWEEPS group compared to the PIPS or CONV group.

A study conducted by Shu Wan et al. showed that PIPS used in combination with MTAD increases the bond strength of a fiber post [56].

The removal of the smear layer also plays an important role in the retention of a fiber post. A study conducted by M. Mancini et al. demonstrated the superiority of the PIPS and SWEEPS protocols in the removal of the smear layer in comparison to ultrasonic and syringe irrigation [28]. Also, a study conducted by Y. Ozbay et al. demonstrated the superiority of PIPS in smear layer removal, with PIPS being superior to Er,Cr:YSGG and Nd:YAG Laser [43]. Nair MR et al. also came to the conclusion, that PIPS protocol is efficient for root canal debridement [59]. The study of Mochizuki S. et al. also showed that PIPS compared to conventional irrigation was satisfactory in smear layer removal while also conserving the dentinal walls [60]. Liu H. et al. concluded that Er:YAG laser has the capacity to dissolute the smear layer at a faster rate than conventional irrigation methods, comparing it also to CleanFlow and Er,Cr:YSGG [61].

After searching the literature, no studies evaluating the effect of SWEEPS on the bond strength of a fiber post could be found. A study conducted by Mancini et al. states that the SWEEPS technique can improve the cleaning and disinfecting efficacy of PIPS technique, which was confirmed in the present study [28].

Despite the generally good results obtained in other studies concerning laser-assisted irrigation on the topic of fiber post retention, the number of studies conducted is still low. This is especially true for the PIPS and SWEEPS protocols. Previous studies have been carried out on extracted teeth, either bovine or human. In vivo studies based on this topic are practically impossible to realize. Still, in the future, the aim should focus on finding study models that can be performed directly in the oral cavity, as those would be the closest to reality. Another factor could be the implication of the Nd:YAG laser concerning the effect on the retention of a fiber post, potentially including an analysis of the dentin surface after the use of a laser. Another factor to consider would be the evaluation of the different root sections. Further studies are necessary to validate the LAI and PIPS techniques, as well as the newly developed SWEEPS protocol, as innovative technologies in modern endodontics.

## 5. Conclusions

The SWEEPS and PIPS protocols resulted in considerably higher fiber post retention than the conventional method, and the SWEEPS protocol was superior to the PIPS protocol.

## Figures and Tables

**Table 1:** The distribution of samples in the experimental groups.

*N Teeth*	*N Samples*	*Pull-Out Test*		*Push-Out Test*	
*48*	96	CONV	16	CONV	16
		PIPS	16	PIPS	16
		SWEEPS	16	SWEEPS	16

**Table 2:** ANOVA test applied to the pull-out test, for all three groups.

	N	Mean	StDev	*p*
CONV Type A (N)	16	93.22	23.82	*p* < 0.05
PIPS Type A (N)	16	113.91	27.51	
SWEEPS Type A (N)	16	150.72	38.57	

**Table 3:** ANOVA test applied to the shear test, for all three groups.

	N	Mean	StDev	*p*
CONV Type B (MPa)	16	8.300	5.642	*p* < 0.05
PIPS Type B (MPa)	16	11.119	4.334	
SWEEPS Type B (MPa)	16	13.456	4.294	

## Data Availability

The raw data supporting the conclusions of this article will be made available by the authors on request.
